# Morphological Diversity, Germplasm Characterization, and Selection Index Analysis of Husk Tomato (*Physalis ixocarpa* Brot.) from Oaxaca, Mexico

**DOI:** 10.3390/plants15091337

**Published:** 2026-04-28

**Authors:** Mabiel Reyes-Fuentes, Enrique González-Pérez, Mariano Mendoza-Elos, Mario Martin González-Chavira, Salvador Villalobos-Reyes, Carlos Alberto Núñez-Colín, Juan Gabriel Ramírez-Pimentel

**Affiliations:** 1 Departamento de Postgrado, Campus Roque, Tecnológico Nacional de México, Celaya 38110, GTO, Mexico; d24980103@roque.tecnm.mx (M.R.-F.); juan.rp1@roque.tecnm.mx (J.G.R.-P.); 2 Programa de Hortalizas, Campo Experimental Bajío, Instituto Nacional de Investigaciones Forestales, Agrícolas y Pecuarias, Celaya 38110, GTO, Mexico; gonzalez.mario@inifap.gob.mx (M.M.G.-C.); villalobos.salvador@inifap.gob.mx (S.V.-R.); 3 Programa de Ingeniería en Biotecnología, División de Ciencias de la Salud e Ingenierías, Universidad de Guanajuato, Celaya 38060, GTO, Mexico; carlos.nunez@ugto.mx

**Keywords:** germplasm evaluation, morphological traits, breeding relevance, selection index

## Abstract

Husk tomato (*Physalis ixocarpa* Brot.) is a crop of major economic, cultural, and nutritional importance in Mexico and exhibits substantial genetic and morphological diversity. Characterizing this variability is essential for both germplasm conservation and breeding programs. During the spring–summer 2024 growing season, 28 husk tomato populations were evaluated at the Bajío Experimental Station (INIFAP), Guanajuato, Mexico, using a completely randomized design with 12 replications. Forty-one traits were assessed following UPOV and IPGRI descriptors. Cluster analysis, canonical discriminant analysis, and the ESIM selection index were applied. A total of 77 morphotypes were identified, exhibiting variation in 33 of the 41 evaluated traits, mainly related to growth habit, leaf morphology, fruit traits, and calyx attributes. Correspondence analysis revealed a close relationship between vegetative growth and fruit size. Cluster analysis clustered the morphotypes into six clusters with no clear geographic structure, suggesting extensive gene flow. Canonical discriminant analysis explained 94.65% of the total variation, identifying seed size, leaf dimensions, and number of anthers as key discriminant traits. The ESIM index highlighted six morphotypes with favorable agronomic and morphological combinations. These results provide a practical basis for the selection of parental materials in husk tomato breeding programs under diverse agroecological conditions.

## 1. Introduction

Husk tomato (*Physalis ixocarpa* Brot. ex Hornem.) is an ancient crop native to Mesoamerica, a region that harbors the greatest diversity of the genus *Physalis*, which comprises approximately 65 species. Mexico is widely recognized as the center of origin, domestication, and diversification of this genus [1]. Although around 15 *Physalis* species are used for human consumption, only *P. ixocarpa*, *P. philadelphica*, and *P. angulata* are extensively cultivated due to their superior fruit quality [2]. Consequently, husk tomato plays a key role in the Mexican diet and cultural heritage [3].

In Mexico, the agricultural relevance of husk tomato is reflected in a cultivated area of 37,504.08 ha, with a total production of 694,327.31 tons and an economic value of USD 255,053,947. The states of Sinaloa, Zacatecas, and Jalisco account for 41.84% of national production, which is predominantly based on traditional landrace materials [4]. Beyond its economic and cultural importance, husk tomato has high nutritional value, as its fruits are a source of minerals, dietary fiber, proteins, and bioactive compounds such as phenolics, carotenoids, and anthocyanins, which are associated with high antioxidant activity and confer a nutraceutical profile of interest for human nutrition and functional food development [5].

Accordingly, numerous studies have focused on characterizing variability in cultivated species, as such diversity constitutes a fundamental pillar for genetic improvement and the conservation of plant genetic resources [6]. In husk tomato, considerable morphological and agronomic variability has been documented in both cultivated germplasm and wild populations [7]. This variability is associated with adaptation to diverse production systems [8], environmental conditions [9], degree of domestication [10], ethnobotanical uses [11], and the species’ complex reproductive biology [12]. Collectively, these factors support the existence of a broad genetic base that represents significant potential for use in breeding programs [13,14].

Phenotypic diversity in husk tomato is expressed primarily in fruit shape and size; however, traits such as plant and fruit biomass, as well as fruit longitudinal and transverse diameters—attributes with high heritability—are considered key selection criteria in breeding processes [15,16,17,18,19]. In addition, wild and cultivated genotypes exhibit contrasting responses under different production systems (e.g., greenhouse versus open-field conditions), highlighting the influence of genotype × environment interactions on yield and on key traits such as fruit number per plant, fruit weight, and equatorial and polar diameters [8,13,20].

Furthermore, several studies indicate that cultivated husk tomato germplasm retains a substantial proportion of the diversity found in wild populations, suggesting a diffuse domestication process [21,22]. This pattern may be explained by the distribution of variability both within and among populations, as well as by the influence of pollinator behavior, dispersal mechanisms, and geographic discontinuities on the genetic structure of the species [23].

The identification of desirable traits—such as plant architecture, fruit size and color, firmness, disease resistance, and yield—enables breeders to select superior genotypes for the development of new varieties [3]. Moreover, morphological characterization facilitates the integration and interpretation of relationships among phenotype, genotype, and agronomic performance. Previous studies have shown that traits such as leaf width and flower diameter are greater in autotetraploid materials than in diploids, although the former often exhibit reduced plant height, underscoring the value of morphological traits as sensitive indicators in advanced breeding programs [24].

Collectively, these studies support germplasm conservation efforts and provide a phenotypic foundation for the selection of genotypes with high yield potential, quality, and adaptation to biotic and abiotic stresses [6,25]. When complemented with visual mass selection, maternal half-sib family selection, and plant-to-plant crossing schemes, these approaches enable the development of improved materials adapted to changing production systems and environmental conditions [3].

Therefore, the objective of this study was to characterize the expression and variability of morphological traits in husk tomato populations evaluated under the agroclimatic conditions of Celaya, Guanajuato, Mexico, in order to identify key traits contributing to phenotypic differentiation and to support conservation and breeding strategies.

## 2. Results

### 2.1. Morphological Characterization

**Figure 1:**
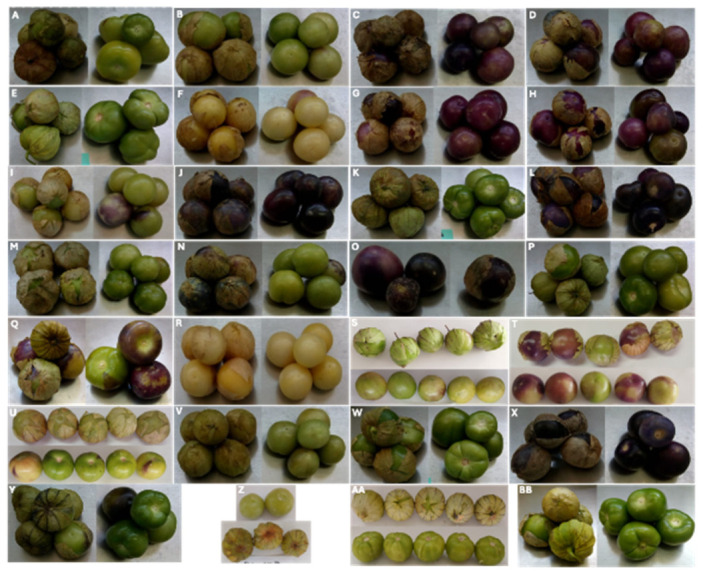
Representative scheme of fruit diversity in husk tomato (*Physalis ixocarpa*), showing the main morphotypes evaluated in this study. (2)-OAX-EDC (**A**), (3)-OAX-SMS (**B**), (4)-OAX-SCP (**C**), (4)-OAX-SSDM (**D**), (6)-OAX-SME (**E**), (7)-OAX-SMD (**F**), (8)-OAX-CDH (**G**), (8)-OAX-SME (**H**), (9)-OAX-SBM (**I**), 10-OAX (**J**), (10)-OAX-TVDM (**K**), (12)-OAX-TDM (**L**), (12)-OAX-TVDM (**M**), (13)-OAX-HDL (**N**), (14)-OAX-LTO (**O**), (16)-OAX-SCP (**P**), (16)-OAX-EDC (**Q**), (17)-OAX-SME (**R**), (22)-OAX-SAS (**S**), (23)-OAX-TDP (**T**), (24)-OAX-TDP (**U**), (26)-OAX-TSL (**V**), (27)-OAX-TVDM (**W**), (34)-OAX-TVDM (**X**), (37)-OAX (**Y**), TV-MEDM (**Z**), TV-CEBAJ (**AA**), TCV-24 (**BB**).

The morphological characterization revealed substantial diversity within the evaluated germplasm. A total of 77 morphotypes were identified among the 28 analyzed populations (Figure 1), exhibiting variation in 33 of the 41 evaluated morphological traits. The greatest variability was observed in traits related to growth habit (GH), intensity of anthocyanin coloration of internodes (IACI), leaf blade shape and margin dentation (LBS and LBDM), leaf blade intensity of green color (LBIGC), petiole attitude and length (PA and PL), and pedicel attitude (FAP). Considerable variation was also detected in fruit-related traits, including fruit size (FS), fruit shape in longitudinal section (FSLS), depth of the stalk cavity (FDSC), main color at harvest maturity (FMCHM), flesh color (FCF), fruit adherence calyx (FAC) and calyx intensity of anthocyanin coloration (CIAC) (Table 1).

In contrast, the absence of pubescence in internodes (PI) and calyx (CP) was consistent across all morphotypes, indicating stability for these traits.

At the plant level, anthocyanin coloration of the hypocotyl (ACH) was observed in 96.2% of the morphotypes. Regarding growth habit, 21.6% of the morphotypes exhibited an upright habit, 68.8% were semi-upright, and 10.3% showed a prostrate growth habit.

Stem traits showed wide variation. Height at first branching (GFB) ranged from 5.1 cm in morphotype (24)-OAX-TDP-MT-2 to 10.4 cm in (34)-OAX-TVDM-MT-1, while internode length (LI) varied from 1.3 cm in (12)-OAX-TDM-MT-1 to 8.4 cm in (24)-OAX-TDP-MT-2 and TV-MEDM-MT-2. Anthocyanin coloration of internodes (ACI) was present in 77.9% of the morphotypes, whereas it was absent in the remainder. The intensity of anthocyanin coloration (IACI) was classified as weak in 33.7% of morphotypes, medium in 36.3%, strong in 7.7%, and absent in 22.1%.

Leaf traits were also highly variable. Medium elliptic leaf blade shape predominated (58.4%), followed by broad elliptic (32.4%) and narrow elliptic forms (9.1%). Leaf blade length (LBL) ranged from 3.2 cm in (10)-OAX-TVDM-MT-2 to 6.6 cm in (26)-OAX-TSL-MT-3, while blade width (LBW) varied from 1.9 cm in (12)-OAX-TDM-MT-4 to 7.1 cm in TV-CEBAJ-23-MT-1. Medium margin dentation (LBDM) was detected in 61.1% of morphotypes. Leaf blade color was predominantly green (64.9%), and medium intensity of green color (LBIGC) was most frequent (70.7%), followed by weak intensity (25.9%).

Petiole attitude was predominantly intermediate (68.8%), while petiole length (PL) ranged from 1.9 cm in (24)-OAX-TDP-MT-2 and (2)-OAX-EDC-MT-1 to 6.7 cm in (26)-OAX-TSL-MT-4.

Flower traits showed marked variability. Flower diameter (FDF) ranged from 1.2 cm in several morphotypes (e.g., (26)-OAX-TSL-MT-1 and (13)-OAX-HDL-MT-3) to 3.1 cm in (10)-OAX-TVDM-MT-1. Five anthers predominated across morphotypes, with only two morphotypes, (6)-OAX-SME-MT-3 and (34)-OAX-TVDM-MT-2, presenting four anthers. Pedicel attitude (FAP) was variable, although an intermediate orientation was most frequent (72.7%).

Fruit traits exhibited pronounced diversity. Small (55.8%) and medium-sized fruits (36.3%) predominated. Fruit length (FL) ranged from 0.9 cm in (12)-OAX-TVDM-MT-3 to 4.17 cm in (26)-OAX-TSL-MT-2, while fruit diameter (FD) varied from 1.4 cm in (17)-OAX-SME-MT-3 to 4.7 cm in (26)-OAX-TSL-MT-2. The length-to-diameter ratio (RLD) revealed round fruits (RLD ≈ 1) in morphotypes such as (26)-OAX-TSL-MT-1 and (22)-OAX-SAS-MT-6, whereas oblate fruits were observed in (12)-OAX-TVDM-MT-3.

Cordate fruit shape in longitudinal section was most frequent (49.3%), followed by circular (36.3%), oblate (10.3%), and triangular (0.33%) shapes. In cross section, circular fruits predominated (91.2%). Depth of the stalk cavity was shallow in 45.4% of morphotypes, medium in 15.5%, deep in 2.5%, and absent in the remaining accessions. Apex shape was predominantly rounded (93.7%), with a pointed apex observed only in (26)-OAX-TSL-MT-3.

The main fruit color at harvest maturity was predominantly green (66.2%), followed by purple (25.9%) and yellow (7.7%), with mainly light color intensity (59.7%). At physiological maturity, fruits were green (46.7%) or purple (42.8%). Fruit firmness was classified as firm in 56 morphotypes and medium in the remainder. Flesh color was mainly green (45.4%) or purplish-green (22.1%). Two locules were observed in 43 morphotypes, whereas five locules occurred in a limited number of morphotypes, including (12)-OAX-TVDM-MT-3 and TCV-24-RCG-MT-1.

Calyx traits also varied. Calyx adherence was classified as strong in 35.1% of morphotypes, medium in 57.7%, and weak in 14.2%. Calyx enclosure was predominantly slightly open (54.5%), followed by very open (31.1%). Ribbing was absent in 42 morphotypes, while anthocyanin coloration of the calyx was observed in 55 morphotypes, with mainly weak (32.4%) or very weak (18.1%) intensity.

Peduncle length ranged from 0.5 cm in (24)-OAX-TDP-MT-2 and (2)-OAX-EDC-MT-1 to 1.1 cm in (17)-OAX-SME-MT-4. Seed color was predominantly yellow (72.7%), and seed size ranged from 10 mg per 100 seeds in TCV-24-RCG-MT-3 to 77 mg per 100 seeds in (34)-OAX-TVDM-MT-1.

### 2.2. Cluster Analysis

**Figure 2:**
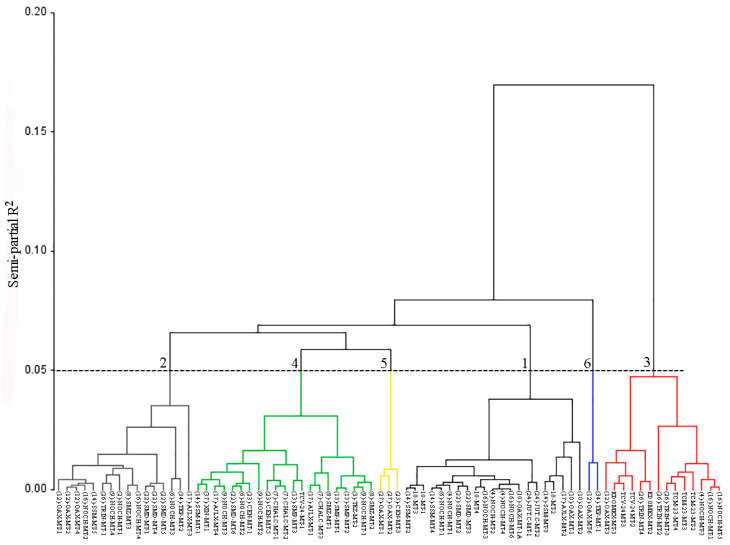
Dendrogram illustrating the morphological similarity among husk tomato morphotypes, generated using Ward’s clustering method.

Cluster analysis grouped the 77 morphotypes into six distinct clusters (Figure 2), each comprising a variable number of morphotypes and sharing their geographical origin to different extents (Table 2). No clear geographic structuring was observed, indicating extensive morphological overlap among populations.

Cluster I consisted of 21 morphotypes originating from multiple localities, including La Trinidad, San Andrés Sinaxtla, San Mateo Etlatongo, and Ejutla de Crespo. This cluster was characterized by a predominance of purple fruits, followed by green and yellow fruits. Cluster II included 16 morphotypes from diverse origins and showed a balanced distribution of green, purple, and yellow fruits.

Cluster III comprised 14 morphotypes originating from Tepelmeme Villa de Morelos, Tezoatlán de Segura y Luna, Santa Cruz Papalutla, and Roque, Guanajuato, with green fruits being predominant. Cluster IV included 21 morphotypes from multiple locations, with green fruits again being most frequent, followed by purple and yellow fruits.

Clusters V and VI were the most compact, containing three and two morphotypes, respectively. These clusters originated exclusively from Tepelmeme Villa de Morelos and/or Tamazulapan de Progreso and were characterized solely by green fruits.

All morphotypes shared seven common traits: anthocyanin coloration of the hypocotyl (ACH), internode pubescence (PI), leaf blade color (LBC), fruit shape in cross section (SCS), apex shape (SA), calyx pubescence (CP), and seed color (SC). Additional traits contributed to cluster differentiation, particularly those related to calyx traits, fruit firmness, and number of locules. Clusters V and VI exhibited the highest internal similarity, sharing 23 traits, while their differentiation was mainly associated with calyx adherence, ribbing, and color intensity traits.

### 2.3. Correlation Analysis

Fruit length (FL) showed a strong and highly significant positive correlation with fruit diameter (FD), indicating a close association between these two variables that describe fruit size. In contrast, the fruit length-to-diameter ratio (RLD) was negatively correlated with fruit diameter, reflecting an inverse relationship between fruit width and fruit shape.

The predominant number of locules (PNL) exhibited low and non-significant correlations with fruit size traits, including FL and FD, indicating a weak association between internal fruit structure and external fruit dimensions.

Among vegetative traits, internode length (LI) was positively correlated with leaf blade length (LBL), leaf blade width (LBW), and flower diameter (FDF). Additionally, flower diameter (FDF) showed positive correlations with leaf size variables and fruit size traits, indicating coordinated variation among vegetative, floral, and fruit characteristics.

Seed size (SS) showed low correlation coefficients with most of the evaluated traits, including fruit and vegetative variables. Similarly, the number of anthers (NA) exhibited weak or non-significant correlations with the majority of agronomic traits.

Overall, the correlation matrix indicated that traits related to fruit size and vegetative growth were more strongly associated, whereas reproductive traits such as seed size, number of anthers, and number of locules exhibited greater independence (Table 3).

### 2.4. Canonical Discriminant Analysis

Canonical discriminant analysis revealed that the first three canonical roots accounted for 94.65% of the total morphological variation among morphotypes (Table 4). The first canonical root (CAN1) explained 69.63% of the variation and was strongly and positively associated with seed size (SS). The second root (CAN2) accounted for 17.41% of the variation and was positively correlated with leaf blade length (LBL), leaf blade width (LBW), and petiole length (PeL). The third root (CAN3), explaining 7.60% of the variation, showed a strong negative association with the number of anthers (NA).

**Figure 3:**
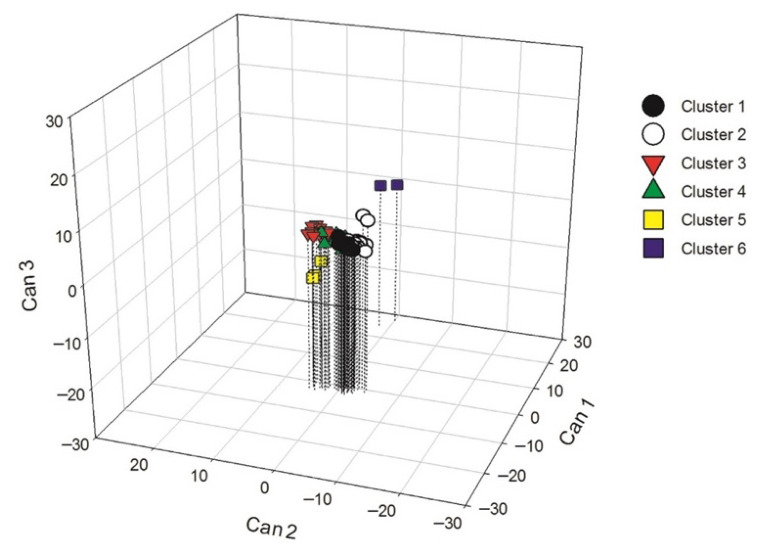
Three-dimensional projection of the 77 husk tomato morphotypes based on the canonical discriminant analysis.

In the three-dimensional projection (Figure 3), morphotypes belonging to Cluster VI showed the highest positive values along CAN1, reflecting larger seed size. In contrast, morphotypes from Cluster V exhibited the lowest CAN1 scores, corresponding to smaller seeds. Along CAN2, Cluster V morphotypes also showed lower scores, associated with reduced leaf size, whereas the remaining clusters were characterized by larger leaves. Along CAN3, clusters were mainly differentiated by variation in the number of anthers and locules.

### 2.5. Selection Index (ESIM)

The eigenanalysis-based selection index (ESIM) incorporated twelve morphological traits with both positive and negative weights. Positive contributions were observed for height at first branching, basal stem diameter, leaf blade length and width, petiole length, fruit length, fruit diameter, and predominant number of locules. In contrast, internode length, number of anthers, seed size, and length-to-diameter ratio exhibited negative weights.

Based on the theoretical ESIM values, six morphotypes were identified as having the highest selection potential (Table 5). Among them, morphotype (26)-OAX-TSL-MT-2 was particularly outstanding, exhibiting a semi-upright growth habit, robust stem, large elliptic leaves, long petiole, flowers with five anthers, slightly open calyx, and large circular fruits with two locules. The remaining selected morphotypes also showed favorable combinations of agronomically relevant traits, highlighting their potential use in breeding programs.

## 3. Discussion

### 3.1. Morphological Characterization

The variation observed in stem, leaf, flower, and fruit traits among the evaluated morphotypes highlights the extensive morphological diversity present within the original husk tomato populations. These results reinforce previous findings indicating that qualitative leaf and fruit traits are effective for differentiating husk tomato morphotypes or populations, even when overall agronomic characteristics are highly similar [26]. The lack of variation in certain traits—such as anthocyanin coloration of the hypocotyl (ACH), internode pubescence (PI), leaf blade color (LBC), fruit shape in cross section (SCS), apex shape (SA), calyx pubescence (CP), and seed color (SC)—within populations suggests a high degree of uniformity for these attributes.

Nevertheless, variation in fruit color was detected within populations, which may be attributed to cross-pollination promoted by the high level of self-incompatibility characteristic of husk tomato [10]. Consistent with previous reports, collections, accessions, lines, or varieties shared common morphological traits regardless of their geographic origin [7,26], a pattern that was also confirmed in the present study. This supports the notion that geographic origin alone is not a reliable predictor of phenotypic differentiation in this species.

Each morphotype exhibited distinctive traits that allowed its differentiation and are relevant for selection processes [27]. The wide variability in fruit size observed in this study is consistent with previous reports for native husk tomato materials, in which polar fruit diameter ranges from 2.31 to 4.61 cm and equatorial diameter from 2.47 to 5.53 cm [28]. In contrast, improved materials typically exhibit larger fruit dimensions [6,29]. The variation in fruit size detected here was closely associated with plant growth habit, confirming the importance of plant architecture in determining yield-related traits.

Divergence in fruit shape among morphotypes was also evident. Several morphotypes exhibited length-to-diameter ratio (RLD) values close to 1, indicative of round fruits [30], whereas oblate and cordate shapes were less frequent. In addition, most germplasm evaluated—including accessions, improved lines, and local varieties derived from landrace materials—showed variation in the number of locules, irrespective of fruit size [31], which agrees with previous observations.

Overall, the morphological characterization of the 77 morphotypes underscores the relevance of growth habit (GH), intensity of anthocyanin coloration of internodes (IACI), leaf blade shape (LBS), margin dentation (LBDM), leaf blade intensity of green color (LBIGC), petiole attitude (PA), pedicel attitude (FAP), fruit size (FS), fruit shape in longitudinal section (FSLS), depth of stalk cavity (FDSC), main color at harvest maturity (FMCHM), flesh color (FCF), calyx adherence (AC), and intensity of calyx anthocyanin coloration (CIAC) as key attributes for husk tomato morphological characterization. In this context, reliance exclusively on quantitative traits—traditionally focused on yield and fruit size—is insufficient for differentiating collections, genotypes, and varieties [28,32]. Therefore, incorporating qualitative traits alongside quantitative descriptors is essential to strengthen characterization efforts and support domestication and breeding strategies.

### 3.2. Cluster Analysis

The dendrogram structure derived from cluster analysis enabled the identification of well-defined groups based on morphological similarity among morphotypes [27,33]. This approach facilitated the objective determination of the optimal number of clusters and their hierarchical relationships [25]. The resulting classification revealed the coexistence of morphotypes from different geographic regions within the same clusters, indicating substantial phenotypic overlap among populations.

Each branch of the dendrogram reflected the relationships among clusters, allowing the identification of patterns of variability and affinity, as well as relative distances among morphotypes. The number of morphotypes per cluster depended on the type and amount of germplasm evaluated, as previously reported in clustering studies based on collections [28], populations [7], and accessions [28]. While many clustering studies rely primarily on quantitative traits [28], such approaches may underestimate the contribution of qualitative descriptors. The present study demonstrated that qualitative traits provide complementary and relevant information for delimiting divergent clusters.

Cluster I was mainly characterized by traits associated with a semi-upright plant ideotype, including anthocyanin coloration of internodes (ACI), number of anthers (NA), fruit firmness (FIRM), and number of locules (PNL). This plant architecture is generally preferred by growers because it allows higher planting densities and, consequently, increased yield potential [7]. However, the morphotypes included in this cluster predominantly produced small to medium-sized fruits, which may limit their suitability for breeding programs focused on current market preferences [13].

In contrast, Clusters V and VI, despite comprising a smaller number of morphotypes, exhibited a higher proportion of favorable traits. These clusters were characterized by taller plants with thicker stems, compact canopies, and green, firm fruits of medium to large size. Such attributes position these morphotypes as promising candidates for incorporation into genetic improvement programs [28,31]. Similar patterns of variability have been reported in both cultivated and wild germplasm of *Physalis philadelphica*, particularly for traits related to fruit size, color, firmness, and growth habit [7,10].

The absence of clear geographic structuring among clusters suggests extensive gene flow among collection sites, likely facilitated by human seed exchange and dispersal mechanisms involving animals and pollinators [23].

### 3.3. Correlation Analysis

The correlation analysis revealed a clear integration among several morphological traits, together with a relative independence among others. The strong positive correlation between fruit length (FL) and fruit diameter (FD) indicates that fruit size in *Physalis ixocarpa* results from coordinated growth along both longitudinal and transverse axes. This pattern is consistent with previous reports in *Physalis* and other Solanaceae, which describe a shared developmental control of fruit growth and expansion processes [3,34].

The negative correlation between the fruit length-to-diameter ratio (RLD) and fruit diameter suggests that, as fruit size increases, fruits tend to adopt a more rounded shape, associated with proportionally greater transverse growth. This morphological trend has been previously described in tomato and tomatillo and reflects shifts in growth allocation during fruit development [35].

In contrast, the absence of a significant correlation between the predominant number of locules (PNL) and fruit size traits indicates that internal fruit structure is largely independent of external fruit dimensions. This behavior, which differs from that observed in more intensively domesticated species, has been attributed in *Physalis* to a diffuse domestication process and high intra-population variability [10,21].

Positive correlations among internode length, leaf size, and flower diameter reflect a close association between vegetative vigor and reproductive development. Likewise, the positive relationships between leaf size, flower diameter, and fruit size support a functional source–sink dynamic, whereby greater photosynthetic capacity is associated with enhanced fruit development [8,36].

Finally, the low correlations of seed size and number of anthers with most vegetative and fruit traits suggest a more autonomous regulation of these reproductive characteristics, as previously reported for *Physalis* [12,37]. Overall, these results confirm that vegetative traits and fruit size form a functionally integrated system, whereas other reproductive traits display greater independence, with important implications for morphological characterization and selection in *Physalis ixocarpa*.

### 3.4. Canonical Discriminant Analysis

Canonical discriminant analysis allowed the identification of traits contributing most strongly to the differentiation among morphotypes along the three canonical axes [38,39]. In the present study, morphological variability was primarily determined by leaf traits (leaf blade length and width), flower traits (number of anthers), and seed traits (seed size), all of which are highly relevant for their incorporation into breeding programs.

These findings are consistent with previous studies identifying traits such as height at first branching, fruit diameter, fruit color, and growth habit as key criteria for population differentiation [7,28]. Consequently, relying exclusively on yield-based comparisons among populations, collections, or improved materials may lead to incomplete or misleading interpretations of genetic diversity [28]. Moreover, canonical discriminant analysis suggested that geographic origin may influence variability patterns, although it does not strictly determine cluster structure [26,32].

### 3.5. Selection Index

The application of the eigenanalysis-based selection index (ESIM) proved effective in identifying morphotypes with superior breeding potential, highlighting six materials [(26)-OAX-TSL-MT-2, (26)-OAX-TSL-MT-3, 10-OAX-MT-2, (10)-OAX-TVDM-MT-1, TV-CEBAJ-23-MT-2, and TV-CEBAJ-23-MT-3] that combined favorable expressions of multiple agronomically relevant traits. Rather than excelling in a single characteristic, these morphotypes exhibited a well-balanced integration of plant architecture, vegetative vigor, reproductive attributes, and fruit traits associated with yield and market quality.

Traits with the greatest contributions to the first eigenvector—across the three canonical axes—were primarily related to leaf size, fruit size and shape, and number of locules. These attributes have been consistently reported as key determinants of phenotypic differentiation and productive potential in native Mexican husk tomato germplasm, reinforcing their relevance as indirect selection criteria [28]. In contrast, the negative contributions of variables such as the length-to-diameter ratio, seed size, internode length, and number of anthers suggest that these traits may play a secondary role or reflect trade-offs in the definition of agronomically desirable ideotypes, depending on specific breeding objectives.

Taken together, these findings underscore the need to reorient husk tomato breeding strategies toward a more integrative framework that explicitly accounts for the extensive morphological variability present among morphotypes. This variability reflects both intrinsic biological characteristics of the species [26] and the cumulative effects of farmer selection and management practices. Importantly, such diversity should not be regarded as a limitation but rather as a strategic resource that can be exploited to enhance agronomic performance, fruit quality, and adaptation to diverse and changing environments [31,37].

The effectiveness of the ESIM in discriminating superior morphotypes is consistent with recent studies demonstrating the robustness of multivariate selection indices for the simultaneous improvement of complex traits, particularly in the absence of predefined economic weights [38,40]. In *Physalis ixocarpa*, multicharacter selection strategies have repeatedly proven their capacity to identify genotypes with improved yield and fruit quality, supporting the broader applicability of eigenanalysis-based indices as practical and reliable tools in breeding programs [41].

## 4. Materials and Methods

### 4.1. Site, Plant Material and Experimental Design

The study was conducted during the spring–summer 2024 growing season at the Bajío Experimental Station (CEBAJ) of the National Institute of Forestry, Agricultural and Livestock Research (INIFAP), located at 20°34′47″ N latitude and 100°49′13″ W longitude, at an elevation of 1750 m above sea level, in Celaya, Gto, Mexico. The region has a semi-arid, semi-warm climate (Bs1hw(w)), characterized by summer rainfall and cool winters, with a mean annual temperature ranging from 18.4 to 24 °C and an average annual precipitation of 300–600 mm [42].

**Figure 4:**
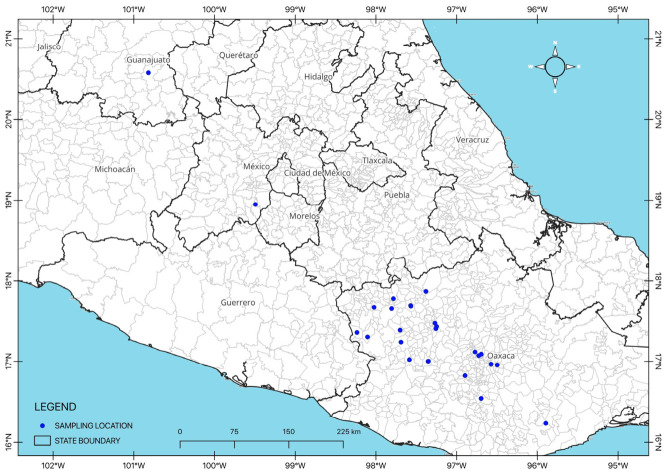
Geographical location of husk tomato sampling sites in Oaxaca, Estado de México, and Guanajuato, México.

The germplasm evaluated belonged to the husk tomato collection of the Vegetable Program at CEBAJ-INIFAP (Table 6) and originated from diverse production areas in the state of Oaxaca. In addition, one population from the State of Mexico and one from Guanajuato were included (Figure 4). A total of 28 populations were evaluated.

Each population was established in plots consisting of five rows, 5.0 m in length and 0.9 m in width, arranged in a double-row zigzag configuration, with a spacing of 30 cm between plants and 25 cm between rows. The experimental design was completely randomized, with 28 treatments (populations) and 12 replications. The experimental unit consisted of an 85 m^2^ plot established on Vertisol soil with a pH of 8.55, organic matter content of 1.68%, bulk density of 0.99 g·cm^−3^, electrical conductivity of 0.9 dS·m^−1^, and nutrient concentrations of 10.55 mg·L^−1^ nitrogen, 18.42 mg·L^−1^ phosphorus, and 399.56 mg·L^−1^ potassium.

Seedlings from each population were produced in polystyrene trays using a peat–vermiculite substrate and maintained for 40 days in a macro tunnel under controlled conditions (28 ± 2 °C and 60 ± 5% relative humidity). During this stage, light irrigation was applied twice daily, and preventive fungicide treatments (copper oxychloride and benomyl at 2.0 g·L^−1^) were applied. Crop management followed the technological package recommended by INIFAP [28].

### 4.2. Data Collected

Morphological characterization was performed using the descriptors of the International Union for the Protection of New Varieties of Plants (UPOV) [43] and the Illustrated Guidelines for the Description of Husk Tomato (*Physalis ixocarpa* Brot. ex Horm.) Varieties [31]. A total of 41 morphological traits were evaluated in plants from each population.

The assessed descriptors were grouped as follows:

- Plant: anthocyanin coloration of the hypocotyl (ACH) and growth habit (GH).
- Stem: height at first branching (GFB), internode length (LI), anthocyanin coloration of internodes (ACI), intensity of anthocyanin coloration of internodes (IACI), and internode pubescence (PI).
- Leaf: blade shape (LBS), blade length (LBL), blade width (LBW), margin dentation (LBDM), blade color (LBC), and intensity of green coloration (LBIGC).
- Petiole: attitude (PA) and length (PL).
- Flower: flower diameter (FDF), number of anthers (NA), and pedicel attitude (FAP).
- Fruit: fruit size (FS), length (FL), diameter (FD), length-to-diameter ratio (RLD), shape in longitudinal section (FSLS), shape in cross section (SCS), depth of the stalk cavity (FDSC), apex shape (SA), main color at harvest maturity (FMCHM), intensity of the main color at harvest maturity (IMCHM), main color at physiological maturity (MCPM), firmness (FIRM), flesh color (FCF), and predominant number of locules (PNL).
- Calyx: pubescence (CP), calyx adherence (AC), calyx enclosure (EC), ribbing (CR), anthocyanin coloration (CAC), and intensity of anthocyanin coloration (CIAC).
- Peduncle: length (PeL).
- Seed: color (SC) and size (SS).

Each trait was scored according to the corresponding UPOV descriptor states.

### 4.3. Statiscal Analysis

Based on the information derived from the morphological characterization, the proportion of morphotypes sharing similar trait expressions was estimated. To assess patterns of similarity and dissimilarity among morphotypes, cluster analysis was performed using quantitative variables: fruit length (FL), fruit diameter (FD), length-to-diameter ratio (RLD), predominant number of locules (PNL), seed size (SS), height at first branching (GFB), internode length (LI), number of anthers (NA), leaf blade length (LBL), leaf blade width (LBW), and peduncle length (PeL). Ward’s clustering method was applied using Euclidean distance [44].

Pearson correlation and Canonical discriminant analysis (CDA) were subsequently conducted using the same set of quantitative variables, with the clusters defined by the dendrogram used as the classification criterion [24]. Finally, the theoretical selection value of each morphotype was estimated using the Eigenanalysis-based selection index (ESIM) proposed by Cerón-Rojas et al. [38].

All statistical analyses were performed using the Statistical Analysis System (SAS) software, version 9.1 [45].

## 5. Conclusions

Husk tomato (*Physalis ixocarpa* Brot.) germplasm exhibited substantial morphological diversity, as demonstrated by the identification of 77 morphotypes grouped into six well-defined clusters, independent of geographic origin. Vegetative traits were closely associated with fruit size, whereas reproductive traits showed greater independence. Seed size, leaf morphology, and number of anthers were the main drivers of morphotype differentiation, and the eigenanalysis-based selection index ESIM identified six morphotypes with superior agronomic potential, supporting their use in breeding programs.

## Figures and Tables

**Table 1:** Agronomically relevant morphological variation among husk tomato accessions evaluated during the spring–summer 2024 growing season in Celaya, Guanajuato, Mexico.

Morph Type	GH	IACI	LBS	LBDM	LBIGC	PA	FAP	FS	FSLS	FDSC	FMCHM	FCF	AC	CIAC
(12)-OAX-TDM-MT-1 *	Up	St	ME	Me	We	S-Up	Int	Sm	Co	Sh	Pu	PG	St	VW
(12)-OAX-TDM-MT-4	S-Up	Me	ME	Me	Me	Int	Int	Sm	Ci	Sh	Pu	Gr	Me	VW
(12)-OAX-TVDM-MT-3	S-Up	Me	ME	Me	Me	Int	Int	Sm	Ci	Ab	Gr	Gr	St	We
(26)-OAX-TSL-MT-1	S-Up	We	ME	Me	We	Int	Int	Sm	Ci	Sh	Gr	Pu	St	Me
(26)-OAX-TSL-MT-2	S-Up	Me	BE	Me	Me	S-Up	Int	VL	Co	Sh	Gr	Gr	Me	We
(26)-OAX-TSL-MT-3	S-Up	We	BE	Me	Me	Int	Int	Me	Co	Me	Gr	Ye	Me	We
(26)-OAX-TSL-MT-4	Up	We	BE	St	We	Int	Int	Sm	Ci	Ab	Gr	Gr	We	VW
(22)-OAX-SAS-MT-1	Pr	Me	ME	Me	Me	Int	Int	Sm	Co	Sh	Ye	Ye	Me	VW
(22)-OAX-SAS-MT-6	S-Up	St	ME	Me	Me	Int	Int	Me	Tr	Sh	Ye	Ye	Me	VW
(17)-OAX-SME-MT-3	Up	Me	ME	Me	Me	Dr	Int	Sm	Ci	Ab	Gr	GY	We	VW
(17)-OAX-SME-MT-4	S-Up	We	ME	Me	Me	Int	Int	Me	Co	Me	Gr	GY	St	We
10-OAX-MT-1	Up	Me	BE	Me	Me	Int	Int	Sm	Co	Me	Gr	Gr	St	We
10-OAX-MT-2	S-Up	We	ME	Me	St	Dr	Int	La	Co	Me	Gr	Gr	St	We
(23)-OAX-TDP-MT-5	S-Up	We	ME	We	Me	S-Up	Int	Me	Ob	Me	Gr	Gr	St	We
(24)-OAX-TDP-MT-2	S-Up	We	ME	Me	Me	Int	Int	Me	Ci	Sh	Pu	Ye	Me	We
(13)-OAX-HDL-MT-1	Up	We	ME	We	We	Int	Int	Sm	Ci	Ab	Gr	GY	Me	VW
(13)-OAX-HDL-MT-3	S-Up	We	BE	Me	We	Int	Int	Sm	Ci	Sh	Gr	Gr	We	VW
(8)-OAX-CDH-MT-1	S-Up	Me	ME	Me	Me	Int	Int	Sm	Ob	Ab	Gr	Gr	Me	We
(2)-OAX-EDC-MT-1	S-Up	Me	NE	We	Me	S-Up	Int	Sm	Tr	Ab	Gr	GY	Me	VW
(6)-OAX-SME-MT-3	S-Up	We	ME	We	Me	Int	Int	Sm	Ci	Ab	Gr	Pu	We	VW
(10)-OAX-TVDM-MT-1	S-Up	We	BE	Me	Me	Int	Dr	La	Co	Sh	Gr	Gr	Me	VW
(10)-OAX-TVDM-MT-2	S-Up	We	BE	Me	Me	Int	Int	Me	Ob	Sh	Gr	Gr	St	Me
(34)-OAX-TVDM-MT-1	S-Up	We	NE	We	Me	Int	Int	La	Ci	Sh	Gr	Gr	St	We
(34)-OAX-TVDM-MT-2	S-Up	Me	ME	St	Me	Int	Int	Sm	Ci	Me	Gr	Gr	St	VW
TV-CEBAJ-23-MT-1	Up	We	ME	St	We	S-Up	Int	Sm	Ci	Sh	Gr	Gr	St	We
TV-CEBAJ-23-MT-2	S-Up	Me	ME	Me	We	Int	Int	Me	Co	Ab	Gr	Gr	Me	Me
TCV-24-RCG-MT-1	S-Up	We	NE	We	Me	S-Up	Int	Me	Ci	Ab	Gr	Gr	St	VW
TCV-24-RCG-MT-3	Up	We	BE	St	Me	Int	Int	Sm	Ob	Sh	Gr	Gr	St	VW

Growth habit (GH), intensity anthocyanin coloration of internodes (IACI), leaf blade shape (LBS), leaf blade dentation of margin (LBDM), leaf blade intensity of green color (LBIGC), petiole attitude (PA), attitude of pedicel (FAP), fruit size (FS), fruit shape in longitudinal section (FSLS), depth of stalk cavity (FDSC), fruit main color at harvest maturity (FMCHM), fruit color of flesh (FCF), adherence calyx (AC), calyx intensity of anthocyanin coloration (CIAC), upright (Up), semi-upright (S-Up), prostrate (Pr), strong (St), weak (We), medium (Me), medium elliptic (ME), broad elliptic (BE), narrow elliptic (NE), intermediate (Int), drooping (Dr), small (Sm), large (La), very large (VL), cordate (Co), circular (Ci), oblate (Ob), triangular (Tr), shallow (Sh), absent (Ab), purple (Pu), green (Gr), yellow (Ye), purplish green (PG), greenish yellow (GY), very weak (VW), * Full data is available in the Supplementary Material.

**Table 2:** Clustering of 77 husk tomato (*Physalis ixocarpa*) morphotypes derived from 28 original populations based on χ^2^ distance using the UPGMA method.

Cluster	Number	Morphotypes
I	21	(10)-OAX-TVDM-MT-2, (10)-OAX-TVDM-MT-1, (17)-OAX-SME-MT-2, 10-OAX-MT-2, (14)-OAX-LTO-MT-3, (24)-OAX-TDP-MT-2, (24)-OAX-TDP-MT-1, (10)-OAX-TVDM-MT-3, (16)-OAX-SCP-MT-6, (4)-OAX-SSDM-MT-4, (4)-OAX-SSDM-MT-2, (16)-OAX-EDC-MT-3, 10-OAX-MT-4, (22)-OAX-SAS-MT-3, (22)-OAX-SAS-MT-2, (4)-OAX-SCP-MT-1, (6)-OAX-SME-MT-1, (14)-OAX-LTO-MT-4, 10-OAX-MT-1, 10-OAX-MT-3, (14)-OAX-LTO-MT-2
II	16	(17)-OAX-SME-MT-3, (34)-OAX-TVDM-MT-2, (6)-OAX-SME-MT-3, (22)-OAX-SAS-MT-5, (22)-OAX-SAS-MT-4, (22)-OAX-SAS-MT-1, (16)-OAX-SCP-MT-4, (8)-OAX-CDH-MT-3, (2)-OAX-EDC-MT-1, (9)-OAX-SBM-MT-4, (26)-OAX-TSL-MT-1, (14)-OAX-LTO-MT-5, (16)-OAX-EDC-MT-2, (12)-OAX-TDM-MT-4, (12)-OAX-TDM-MT-2, (12)-OAX-TDM-MT-1
III	14	(16)-OAX-EDC-MT-5, (16)-OAX-SCP-MT-1, (4)-OAX-SCP-MT-3, TV-CEBAJ-23-MT-1, TV-CEBAJ-23-MT-2, TV-CEBAJ-23-MT-3, (26)-OAX-TSL-MT-3, (26)-OAX-TSL-MT-2, TV-MEDM-MT-2, (26)-OAX-TSL-MT-4, TCV-24-RCG-MT-2, TCV-24-RCG-MT-3, TV-MEDM-MT-3, (12)-OAX-TVDM-MT-3
IV	21	(8)-OAX-CDH-MT-2, (9)-OAX-SBM-MT-1, (3)-OAX-SMS-MT-2, (13)-OAX-HDL-MT-2, (13)-OAX-HDL-MT-1, (8)-OAX-CDH-MT-1, (7)-OAX-SMD-MT-3, (17)-OAX-SME-MT-1, TCV-24-RCG-MT-1, (13)-OAX-HDL-MT-3, (7)-OAX-SMD-MT-2, (7)-OAX-SMD-MT-1, (23)-OAX-TDP-MT-5, (9)-OAX-SBM-MT-2, (23)-OAX-TDP-MT-1, (6)-OAX-SME-MT-2, (22)-OAX-SAS-MT-6, (9)-OAX-SBM-MT-3, (17)-OAX-SME-MT-4, (37)-OAX-XIN-MT-1, (14)-OAX-LTO-MT-1
V	3	(23)-OAX-TDP-MT-3, (27)-OAX-TVDM-MT-2, (27)-OAX-TVDM-MT-1
VI	2	(34)-OAX-TVDM-MT-1, (12)-OAX-TDM-MT-6

**Table 3:** Pearson’s correlation coefficient among twelve agronomic traits evaluated in 77 husk tomato morphotypes.

	FL	FD	RLD	PNL	SS	GFB	LI	FDF	NA	LBL	LBW
FD	0.924 ***	1									
RLD	0.055	−0.306 **	1								
PNL	−0.035	0.055	−0.215	1							
SS	0.150	0.062	0.165	0.010	1						
GFB	−0.036	−0.066	0.083	0.115	0.015	1					
LI	0.062	0.029	0.016	−0.048	−0.063	−0.094	1				
FDF	0.284 *	0.344 **	−0.208	−0.293 **	−0.083	−0.140	0.118	1			
NA	−0.030	0.011	−0.083	−0.023	−0.015	0.053	−0.165	−0.107	1		
LBL	0.126	0.165	−0.159	−0.053	−0.154	−0.133	0.333 **	0.366 ***	−0.001	1	
LBW	0.143	0.188	−0.200	−0.055	−0.163	−0.182	0.310 **	0.353 **	0.028	0.941 ***	1
PeL	0.103	0.150	−0.143	0.009	−0.172	−0.072	0.297 **	0.370 ***	0.004	0.820 ***	0.748 ***

* *p* ≤ 0.05, ** *p* ≤ 0.01, *** *p* ≤ 0.001. Fruit length (FL; cm), fruit diameter (FD; cm), fruit ratio length/diameter (RLD), fruit predominant number of locules (PNL),seed size (SS; g), height at first branching (GFB; cm), length internodes (LI; cm), flower diameter (FDF; cm), number of anthers (NA), leaf blade length (LBL; cm), leaf blade width (LBW; cm), petiole length (PeL; cm).

**Table 4:** Eigenvalues and canonical discriminant structure of 77 husk tomato morphotypes based on eleven agronomic traits.

Trait	CAN1	CAN2	CAN3
Percentage of variance (%)	69.63	17.41	7.60
Cumulative variance (%)	69.63	87.05	94.65
Fruit length (FL)	0.0517	0.1045	0.0921
Fruit diameter (FD)	−0.0232	0.1937	0.1037
Fruit ratio length/diameter (RLD)	0.1552	−0.2861	−0.0715
Fruit predominant number of locules (PNL)	0.0690	0.0113	0.0302
Seed size (SS)	0.9876 *	0.0276	−0.0136
height at first branching (GFB)	0.0078	−0.0350	−0.2101
Length internodes (LI)	−0.1251	0.2943	0.2792
Flower diameter (FDF)	−0.1580	0.3517	0.4161
Number of anthers (NA)	−0.0426	0.4236	−0.8839
Leaf blade length (LBL)	−0.1804	0.8276	0.3908
Leaf blade width (LBW)	−0.1806	0.7970	0.3614
Petiole length (PeL)	−0.1619	0.8223	0.3662

* Values highly correlated with the canonical root (CAN).

**Table 5:** ESIM selection index applied to 77 husk tomato morphotypes.

Morphotype	SI	Morphotype	SI	Morphotype	SI	Morphotype	SI
(26)-OAX-TSL-MT-2	33.655	(14)-OAX-LTO-MT-4	21.603	(37)-OAX-XIN-MT-1	19.498	(26)-OAX-TSL-MT-4	17.314
10-OAX-MT-1	28.225	(10)-OAX-TVDM-MT-3	21.487	(6)-OAX-SME-MT-2	19.384	(17)-OAX-SME-MT-1	17.288
TV-CEBAJ-23-MT-3	26.870	(6)-OAX-SME-MT-1	21.359	EDOMEX-MT-3	19.353	(14)-OAX-LTO-MT-5	17.2285
TV-CEBAJ-23-MT-2	26.184	TCV-24-RCG-MT-1	21.053	10-OAX-MT-4	19.341	(12)-OAX-TVDM-MT-6	17.031
(26)-OAX-TSL-MT-3	25.780	(22)-OAX-SAS-MT-6	21.032	EDOMEX-MT-2	19.312	(26)-OAX-TSL-MT-1	16.985
10-OAX-MT-2	25.358	(16)-OAX-SCP-MT-6	21.004	(22)-OAX-SAS-MT-2	19.141	(12)-OAX-TVDM-MT-3	16.779
(9)-OAX-SBM-MT-2	24.927	(22)-OAX-SAS-MT-5	20.947	(23)-OAX-TDP-MT-1	19.097	(2)-OAX-EDC-MT-1	16.627
(17)-OAX-SME-MT-2	24.763	(16)-OAX-EDC-MT-3	20.894	(22)-OAX-SAS-MT-3	18.806	(9)-OAX-SBM-MT-1	16.396
(14)-OAX-LTO-MT-3	24.021	(23)-OAX-TDP-MT-3	20.892	(24)-OAX-TDP-MT-1	18.749	(3)-OAX-SMS-MT-2	16.356
(16)-OAX-SCP-MT-1	23.554	(13)-OAX-HDL-MT-3	20.711	10-OAX-MT-3	18.734	(8)-OAX-SME-MT-2	16.308
(23)-OAX-TDP-MT-5	23.096	(24)-OAX-TDP-MT-2	20.601	(9)-OAX-SBM-MT-4	18.554	(12)-OAX-TDM-MT-1	15.934
(17)-OAX-SME-MT-4	22.425	(9)-OAX-SBM-MT-3	20.326	(7)-OAX-SMD-MT-1	18.509	(8)-OAX-SME-MT-3	15.802
TCV-24-RCG-MT-2	22.339	(27)-OAX-TVDM-MT-1	20.223	(34)-OAX-TVDM-MT-2	18.387	(16)-OAX-SCP-MT-4	15.419
TCV-24-RCG-MT-3	22.238	(22)-OAX-SAS-MT-1	20.205	(12)-OAX-TDM-MT-2	18.231	(13)-OAX-HDL-MT-2	15.312
(34)-OAX-TVDM-MT-1	22.219	(14)-OAX-LTO-MT-1	20.058	(27)-OAX-TVDM-MT-2	17.987	(17)-OAX-SME-MT-3	14.921
(4)-OAX-SSDM-MT-4	22.123	(7)-OAX-SMD-MT-2	20.054	(10)-OAX-TVDM-MT-1	17.922	(13)-OAX-HDL-MT-1	14.683
TV-CEBAJ-23-MT-1	21.988	(4)-OAX-SCP-MT-1	20.041	(12)-OAX-TDM-MT-4	17.852	(8)-OAX-CDH-MT-1	13.281
(16)-OAX-EDC-MT-5	21.858	(22)-OAX-SAS-MT-4	19.905	(7)-OAX-SMD-MT-3	17.701		
(4)-OAX-SCP-MT-3	21.851	(10)-OAX-TVDM-MT-2	19.879	(6)-OAX-SME-MT-3	17.539		
(4)-OAX-SSDM-MT-2	21.614	(16)-OAX-EDC-MT-3	19.865	(14)-OAX-LTO-MT-2	17.445		

ESIM selection index (SI).

**Table 6:** Geographical location of the sampling sites of the 28 original populations.

Code	Type	Location	LN	LO	Altitude ^†^
(2)-OAX-EDC	Green	Ejutla de Crespo	16°32′28.3″	96°41′48.7″	1592
(3)-OAX-SMS	Green	San Mateo Sindihul	17°00′4.8″	97°21′15.3″	2104
(4)-OAX-SCP	Purple	Santa Cruz Papalutla	16°57′52.6″	96°34′23.6″	1498
(4)-OAX-SSDM	Purple	San Sebastián del Monte	17°40′19″	98°01′25.3″	2154
(6)-OAX-SME	Green	San Mateo Etlatongo	17°24′26.2″	97°15′25.3″	2096
(7)-OAX-SMD	Yellow	San Martin Durazno	17°18′16.5″	98°6′15″	2104
(8)-OAX-CDH	Purple	Chalcatongo de Hidalgo	17°01′18.2″	97°35′7.4″	2045
(8)-OAX-SME	Purple	Santa María Ecatepec	16°13′59.2″	95°53′42.2″	1478
(9)-OAX-SBM	Green	San Bernardo Mixtepec	16°49′24″	96°53′45.4″	1593
10-OAX	Purple	Ciudad de Oaxaca	17°4′23.23″	96°43′35.0″	2009
(10)-OAX-TVDM	Green	Tepelmeme Villa de Morelos	17°52′8.2″	97°22′51.5″	1586
(12)-OAX-TDM	Purple	Tlacolula de Matamoros	16°57′17.2″	96°29′50.7″	1648
(12)-OAX-TVDM	Green	Tepelmeme Villa de Morelos	17°52′8.2″	97°22′50″	1994
(13)-OAX-HDL	Green	Huajuapan de León	17°46′44.8″	97°47′2.1″	1588
(14)-OAX-LTO	Purple	La Trinidad Oaxaca	17°7′5.5″	96°46′21.1″	1101
(16)-OAX-SCP	Green	Santa Cruz Papalutla	16°57′52.6″	96°34′23.6″	2443
(16)-OAX-EDC	Purple	Ejutla de Crespo	16°32′28.3″	96°41′48.7″	1786
(17)-OAX-SME	Yellow	San Mateo Etlatongo	17°26′4.8″	97°14′54.1″	1457
(22)-OAX-SAS	Green	San Andres Sinaxtla	17°28′35.7″	97°16′8.9″	1886
(23)-OAX-TDP	Purple	Tamazulapan de Progreso	17°41′37″	97°34′13″	2037
(24)-OAX-TDP	Green	Tamazulapan de Progreso	17°41′12.7″	97°34′3.2″	2104
(26)-OAX-TSL	Green	Tezoatlan de Segura y Luna	17°39′25.2″	97°48′22.3″	1586
(27)-OAX-TVDM	Green	Tepelmeme Villa de Morelos	17°52′8.2″	97°22′51″	1437
(34)-OAX-TVDM	Purple	Tepelmeme Villa de Morelos	17°52′8.2″	97°22′51.5″	1647
(37)-OAX	Green	Santiago Xiacuí	17°17′15″	96°25′43″	1741
TV-MEDM	Green	Malinalco *	18°56′53.2″	99°29′37.04″	2104
TV-CEBAJ	Green	Roque, Celaya **	20°34′45.2″	100°49′11.26″	1766
TCV-24	Green	Roque, Celaya **	20°34′45.2″	100°49′11.26″	1766

Latitude north (LN), longitude west (LO). ^†^ m·a.s.l. * Collected in Estado de México. ** Collected in Guanajuato.

## Data Availability

The datasets collected and analyzed for this study are available upon reasonable request.

## Supplementary Materials

The following supporting information can be downloaded at: https://www.mdpi.com/article/10.3390/plants15091337/s1, Table S1: Agronomically relevant morphological variation among husk tomato accessions evaluated during the spring–summer 2024 growing season in Celaya, Guanajuato, Mexico.
