# Supplementary material for: Morphological Diversity, Germplasm Characterization, and Selection Index Analysis of Husk Tomato (Physalis ixocarpa Brot.) from Oaxaca, Mexico
Source: Plants (Basel). 2026 Apr 28;15(9):1337. doi: 10.3390/plants15091337 (PMC13164964; doi:10.3390/plants15091337)
Supplement: Supplementary file 1 [file plants-15-01337-s001.zip › plants-4208024-supplementary.pdf]

Table S1. Agronomically relevant morphological variation among husk tomato accessions evaluated during the spring–summer 2024 growing season in Celaya, Guanajuato, Mexico.

Morph type	GH	IACI	LBS	LBDM	LBIGC	PA	FAP	FS	FSLS	FDSC	FMCHM	FCF	AC	CIAC
(12)-OAX-TDM-MT-1	Up	St	ME	Me	We	S-Up	Int	Sm	Co	Sh	Pu	PG	St	VW
(12)-OAX-TDM-MT-2	S-Up	We	BE	Me	We	Dr	Int	Sm	Ci	Sh	Pu	PG	St	St
(12)-OAX-TDM-MT-4	S-Up	Me	ME	Me	Me	Int	Int	Sm	Ci	Sh	Pu	Gr	Me	VW
(12)-OAX-TVDM-MT-3	S-Up	Me	ME	Me	Me	Int	Int	Sm	Ci	Ab	Gr	Gr	St	We
(12)-OAX-TVDM-MT-6	S-Up	We	ME	Me	Me	S-Up	Int	La	Ci	Ab	Gr	Gr	Me	VW
(26)-OAX-TSL-MT-1	S-Up	We	ME	Me	We	Int	Int	Sm	Ci	Sh	Gr	Pu	St	Me
(26)-OAX-TSL-MT-2	S-Up	Me	BE	Me	Me	S-Up	Int	VL	Co	Sh	Gr	Gr	Me	We
(26)-OAX-TSL-MT-3	S-Up	We	BE	Me	Me	Int	Int	Me	Co	Me	Gr	Ye	Me	We
(26)-OAX-TSL-MT-4	Up	We	BE	St	We	Int	Int	Sm	Ci	Ab	Gr	Gr	We	VW
(14)-OAX-LTO-MT-1	S-Up	Me	ME	Me	Me	Int	Int	Sm	Co	Me	Pu	PG	Me	We
(14)-OAX-LTO-MT-2	Pr	St	BE	Me	Me	Int	Int	Sm	Co	Me	Pu	Gr	St	We
(14)-OAX-LTO-MT-3	S-Up	We	ME	St	Me	Int	Int	Me	Co	De	Pu	Ye	St	Me
(14)-OAX-LTO-MT-4	S-Up	Me	ME	St	Me	S-Up	Dr	Me	Co	Sh	Pu	GY	Me	St
(14)-OAX-LTO-MT-5	S-Up	Me	ME	Me	Me	Int	Int	Sm	Co	Ab	Pu	Pu	Me	We
(22)-OAX-SAS-MT-1	Pr	Me	ME	Me	Me	Int	Int	Sm	Co	Sh	Ye	Ye	Me	VW
(22)-OAX-SAS-MT-2	S-Up	We	ME	We	We	S-Up	Int	Sm	Ob	Ab	Ye	Ye	Me	VW
(22)-OAX-SAS-MT-3	S-Up	We	ME	Me	We	Int	Int	Sm	Ci	Ab	Ye	GY	St	VW
(22)-OAX-SAS-MT-4	S-Up	Me	ME	We	We	Int	Int	Sm	Co	Sh	Ye	Ye	St	We
(22)-OAX-SAS-MT-5	Up	Me	BE	St	Me	Int	Int	Sm	Co	Sh	Ye	Gr	We	We
(22)-OAX-SAS-MT-6	S-Up	St	ME	Me	Me	Int	Int	Me	Tr	Sh	Ye	Ye	Me	VW
(27)-OAX-TVDM-MT-1	S-Up	St	ME	Me	Me	Int	Int	Me	Ci	Ab	Gr	PG	Me	St
(27)-OAX-TVDM-MT-2	Up	St	ME	Me	We	Int	Int	Sm	Ci	Ab	Gr	Ye	Me	VW
(17)-OAX-SME-MT-1	Up	Me	BE	Me	Me	Int	Int	Me	Co	Me	Gr	Gr	Me	VW
(17)-OAX-SME-MT-2	Pr	We	ME	Me	We	S-Up	Int	Me	Ci	Ab	Gr	PG	Me	We
(17)-OAX-SME-MT-3	Up	Me	ME	Me	Me	Dr	Int	Sm	Ci	Ab	Gr	GY	We	VW
(17)-OAX-SME-MT-4	S-Up	We	ME	Me	Me	Int	Int	Me	Co	Me	Gr	GY	St	We
(37)-OAX-XIN-MT-1	S-Up	We	BE	Me	Me	Dr	Int	Me	Co	Sh	Gr	Gr	St	We
10-OAX-MT-1	Up	Me	BE	Me	Me	Int	Int	Sm	Co	Me	Gr	Gr	St	We
10-OAX-MT-2	S-Up	We	ME	Me	St	Dr	Int	La	Co	Me	Gr	Gr	St	We
10-OAX-MT-3	S-Up	Me	BE	St	Me	Int	Int	Sm	Ci	Me	Gr	Gr	Me	VW
10-OAX-MT-4	S-Up	We	ME	Me	We	Int	Int	Sm	Co	Sh	Gr	Gr	St	VW
(23)-OAX-TDP-MT-1	S-Up	St	ME	Me	Me	Int	Int	Sm	Co	Ab	Gr	Gr	Me	We
(23)-OAX-TDP-MT-3	Pr	We	ME	Me	Me	S-Up	Int	Me	Ci	Ab	Gr	PG	Me	We
(23)-OAX-TDP-MT-5	S-Up	We	ME	We	Me	S-Up	Int	Me	Ob	Me	Gr	Gr	St	We
(4)-OAX-SCP-MT-1	Pr	We	NE	We	Me	S-Up	Int	Me	Ci	Sh	Pu	PG	Me	VW
(4)-OAX-SCP-MT-3	Up	We	BE	St	Me	Dr	Int	Me	Co	Ab	Pu	Pu	St	Me
(4)-OAX-SSDM-MT-2	S-Up	Me	ME	We	We	S-Up	Int	Me	Co	Sh	Gr	Pu	Me	VW
(4)-OAX-SSDM-MT-4	S-Up	Me	ME	Me	Me	Int	Int	Me	Co	Ab	Gr	Gr	St	VW
(24)-OAX-TDP-MT-1	Pr	We	ME	Me	Me	Int	Int	Me	Co	Me	Pu	Ye	St	We

(24)-OAX-TDP-MT-2	S-Up	We	ME	Me	Me	Int	Int	Me	Ci	Sh	Pu	Ye	Me	We
(13)-OAX-HDL-MT-1	Up	We	ME	We	We	Int	Int	Sm	Ci	Ab	Gr	GY	Me	VW
(13)-OAX-HDL-MT-2	S-Up	We	BE	Me	Me	S-Up	Int	Sm	Ci	Sh	Gr	Gr	St	VW
(13)-OAX-HDL-MT-3	S-Up	We	BE	Me	We	Int	Int	Sm	Ci	Sh	Gr	Gr	We	VW
(3)-OAX-SMS-MT-2	S-Up	We l	BE	Me	Me	Int	Dr	Sm	Co	Ab	Gr	Gr	Me	We
(8)-OAX-CDH-MT-1	S-Up	Me	ME	Me	Me	Int	Int	Sm	Ob	Ab	Gr	Gr	Me	We
(8)-OAX-SME-MT-2	S-Up	We	ME	Me	Me	Int	Int	Sm	Ci	Sh	Pu	Pu	Me	VW
(8)-OAX-SME-MT-3	Up	Me	BE	We	Me	S-Up	Int	Sm	Ci	Sh	Pu	PG	Me	VW
(2)-OAX-EDC-MT-1	S-Up	Me	NE	We	Me	S-Up	Int	Sm	Tr	Ab	Gr	GY	Me	VW
(7)-OAX-SMD-MT-1	Up	Me	BE	Me	Me	Int	Int	Sm	Ci	Sh	Pu	PG	Me	VW
(7)-OAX-SMD-MT-2	S-Up	Me	BE	Me	Me	Int	Int	Me	Me	Sh	Pu	Pu	We	VW
(7)-OAX-SMD-MT-3	S-Up	Me	ME	Me	We	Dr	Int	Sm	Co	Sh	Pu	PG	Me	We
(6)-OAX-SME-MT-1	S-Up	We	BE	Me	We	Int	Er	Me	Co	Sh	Gr	PG	Me	Me
(6)-OAX-SME-MT-2	S-Up	We	ME	We	Me	Int	Int	Sm	Co	Sh	Gr	PG	Me	Me
(6)-OAX-SME-MT-3	S-Up	We	ME	We	Me	Int	Int	Sm	Ci	Ab	Gr	Pu	We	VW
(10)-OAX-TVDM-MT-1	S-Up	We	BE	Me	Me	Int	Dr	La	Co	Sh	Gr	Gr	Me	VW
(10)-OAX-TVDM-MT-2	S-Up	We	BE	Me	Me	Int	Int	Me	Ob	Sh	Gr	Gr	St	Me
(10)-OAX-TVDM-MT-3	Up	We	ME	St	Me	Int	Int	Me	Ob	Ab	Gr	Gr	We	St
(16)-OAX-SCP-MT-1	S-Up	We	ME	St	Me	Int	Int	Me	Co	Ab	Gr	PG	Me	VW
(16)-OAX-SCP-MT-4	Pr	Me	ME	Me	Me	Int	Int	Sm	Ci	Ab	Gr	GY	St	VW
(16)-OAX-SCP-MT-6	S-Up	We	ME	Me	Me	Int	Int	Sm	Co	Sh	Gr	Gr	Me	Me
(16)-OAX-EDC-MT-2	S-Up	Me	ME	St	Me	Int	Dr	Me	Ci	Me	Pu	PG	St	Me
(16)-OAX-EDC-MT-3	S-Up	Me	ME	Me	We	S-Up	Int	Me	Ci	Sh	Pu	GY	Me	Me
(16)-OAX-EDC-MT-5	S-Up	We	BE	Me	Me	Int	Int	Me	Ob	Ab	Pu	PG	Me	We
(9)-OAX-SBM-MT-1	S-Up	We	NE	Me	Me	Dr	Int	Sm	Ci	Ab	Gr	PG	Me	We
(9)-OAX-SBM-MT-2	S-Up	Me	BE	St	Me	Int	Int	La	Tr	Sh	Gr	PG	Me	Me
(9)-OAX-SBM-MT-3	Up	We	BE	Me	Me	S-Up	Int	Sm	Co	Sh	Gr	Gr	We	VW
(9)-OAX-SBM-MT-4	Pr	We	NE	Me	We	Int	Int	Sm	Ci	Ab	Gr	Pu	Me	VW
TV-MEDM-MT-2	S-Up	We	ME	Me	St	Dr	Int	Sm	Ob	Ab	Gr	Gr	We	VW
TV-MEDM-MT-3	S-Up	We	NE	Me	St	Int	Int	Sm	Ci	Sh	Gr	Gr	We	VW
(34)-OAX-TVDM-MT-1	S-Up	We	NE	We	Me	Int	Int	La	Ci	Sh	Gr	Gr	St	We
(34)-OAX-TVDM-MT-2	S-Up	Me	ME	St	Me	Int	Int	Sm	Ci	Me	Gr	Gr	St	VW
TV-CEBAJ-23-MT-1	Up	We	ME	St	We	S-Up	Int	Sm	Ci	Sh	Gr	Gr	St	We
TV-CEBAJ-23-MT-2	S-Up	Me	ME	Me	We	Int	Int	Me	Co	Ab	Gr	Gr	Me	v
TV-CEBAJ-23-MT-3	Up	Me	ME	Me	We	Int	Int	Sm	Co	Sh	Gr	Gr	St	We
TCV-24-RCG-MT-1	S-Up	We	NE	We	Me	S-Up	Int	Me	Ci	Ab	Gr	Gr	St	VW
TCV-24-RCG-MT-2	S-Up	We	NE	We	Me	Int	Dr	Me	Co	De	Gr	Gr	We	VW
TCV-24-RCG-MT-3	Up	We	BE	St	Me	Int	Int	Sm	Ob	Sh	Gr	Gr	St	VW

Growth habit (GH), intensity anthocyanin coloration of internodes (IACI), leaf blade shape (LBS), leaf blade dentation of margin (LBDM), leaf blade intensity of green color (LBIGC), petiole attitude (PA), attitude of pedicel (FAP), fruit size (FS), fruit shape in longitudinal section (FSLS), depth of stalk cavity (FDSC), fruit main color at harvest maturity (FMCHM), fruit color of flesh (FCF), adherence calyx (AC), calyx intensity of anthocyanin coloration (CIAC), upright (Up), semi-upright (S-Up), prostrate (Pr), strong (St), weak (We), medium (Me), medium elliptic (ME), broad elliptic (BE), narrow elliptic (NE), intermediate (Int), drooping (Dr), small (Sm), large (La), very large (VL), cordate (Co), circular (Ci), oblate (Ob), triangular (Tr), shallow (Sh), absent (Ab), , purple

(Pu), green (Gr), yellow (Ye), purplish green (PG), greenish yellow (GY), very weak (VW), * Full data is available in the Supplementary Material.
